# One‐year clinical outcomes of anticoagulation therapy among Japanese patients with atrial fibrillation: The Hyogo AF Network (HAF‐NET) Registry

**DOI:** 10.1002/joa3.12226

**Published:** 2019-08-16

**Authors:** Kiyohiro Hyogo, Akihiro Yoshida, Motoshi Takeuchi, Kunihiko Kiuchi, Koji Fukuzawa, Mitsuru Takami, Atsushi Kobori, Katsunori Okajima, Michio Odake, Toshio Okada, Akira Shimane, Yasuhiro Kawahara, Junichi Sekiya, Hiroshi Sano, Yasunori Ichikawa, Ken‐ichi Hirata, the HAF‐NET Registry Investigators

**Affiliations:** ^1^ Section of Arrhythmia, Division of Cardiovascular Medicine, Department of Internal Medicine Kobe University Graduate School of Medicine Kobe Japan; ^2^ Kita‐harima Medical Center Sanda Japan; ^3^ Takeuchi Clinic Kobe Japan; ^4^ Kobe City Medical Center General Hospital Kobe Japan; ^5^ Kakogawa Central City Hospital Kakogawa Japan; ^6^ Odake Internal Medicine Cardiology Kobe Japan; ^7^ Okada Clinic Kobe Japan; ^8^ Himeji Cardiovascular Center Himeji Japan; ^9^ Kawahara Internal Medicine Kobe Japan; ^10^ Ooyama kinen Hospital Kobe Japan; ^11^ Kobe Century Memorial Hospital Kobe Japan; ^12^ Ichikawa Internal Medicine Cardiology Kakogawa Japan

**Keywords:** atrial fibrillation, catheter ablation, dementia, direct oral anticoagulants, warfarin

## Abstract

**Background:**

Although anticoagulation therapy could reduce the risk of strokes in patients with atrial fibrillation (AF), large‐scale investigations in the direct oral anticoagulant (DOAC) and AF catheter ablation (CA) era are lacking.

**Methods:**

This study was designed as a prospective, multicenter, observational study and a total of 2113 patients from 22 institutions were enrolled in the Hyogo area.

**Results:**

The mean age and CHADS_2_ score were 70.1 ± 10.8 years old and 1.5 ± 1.1, respectively. The follow‐up period was 355 ± 43 days. CA was performed in 614 (29%) and DOACs were prescribed in 1118 (53%) patients. Ischemic strokes/systemic embolisms (SEs) and major bleeding occurred in 13 (0.6%) and 17 (0.8%) patients, respectively. New onset dementia, hospitalizations for cardiac events, and all‐cause death occurred in eight (0.4%), 60 (2.8%), and 29 (1.4%) patients, respectively. A multivariate analysis demonstrated that persistent AF and the body weight (BW) were associated with ischemic strokes/SEs and major bleeding, respectively (persistent AF: hazard ratio, 9.57; 95%CI, 1.2‐74.0; *P* = .03; BW: hazard ratio, 0.94; 95%CI, 0.90‐0.99; *P* = .02). AFCA history was associated with the cardiac events (hazard ratio, 0.44; 95%CI, 0.20‐0.99; *P* = .04). Age was associated with new onset dementia (hazard ratio, 1.1; 95%CI, 1.0‐1.2; *P* = .03).

**Conclusions:**

In the DOAC and CA era, the incidence of ischemic strokes/SEs, major bleeding and cardiac events could be dramatically reduced in patients with AF. However, some unsolved issues of AF management still remain especially in elderly patients with persistent AF and a low BW.

**Abbreviations**
AF: atrial fibrillation
CA: catheter ablation
DOAC: direct oral anticoagulants

## 1 INTRODUCTION

The number of patients with atrial fibrillation (AF) is increasing at a rapid rate and is expected to reach beyond one million patients in Japan as the population ages.^1^ AF has a major risk of thromboembolisms and heart failure. Several studies reported that AF is also related to new onset dementia.^2^ The annual incidence of cerebral thromboembolisms in AF patients is almost 2%‐4% in Japan and increases with the CHADS_2_ score/CHA_2_DS_2_‐VASc score number.^3^, ^4^ Direct oral anticoagulants (DOACs) have been widely used to prevent cerebral infarctions in patients with AF. The advantages of DOACs over warfarin in reducing cerebral infarctions and bleeding complications have been demonstrated in several randomized clinical trials (RCT).^5^, ^6^, ^7^, ^8^ However, the long‐term outcomes of DOAC use remain unclear in the catheter ablation (CA) era.

AF catheter ablation (AFCA) is widely performed, and some investigations have reported that it is more effective for preventing AF recurrences than medical therapy.^9^, ^10^ CA in patients with heart failure has been reported to be associated with a significantly lower rate of a composite end point of death from any cause or hospitalization for worsening heart failure than medical therapy, while the impact is less in patients without heart failure.^11^, ^12^ Evidence that the mortality improves in patients who undergo CA is still limited. Therefore, it is important to reveal how to select the best treatment of AF based on each patient's background.

AF patients without strokes have been followed by cardiologists in Japan. However, once cerebral vascular events occur, those patients are followed by brain surgeons or neurologists. Therefore, it is difficult for primary care doctors to share the events. To share those events, we established the HAF‐NET (HYOGO ATRIAL FIBRILLATION NETWORK) registry, which pooled the data from primary care doctors, brain surgeons, and neurologists. The primary end point was the composite of symptomatic cerebral infarctions including transient ischemic attacks (TIAs), systemic embolisms (SEs), and fatal bleeding complications requiring hospitalization including an intracranial hemorrhage. Secondary end points included a composite of a composite of new onset dementia, cardiac events requiring hospitalization, and all‐cause death. Our goal of this study was to examine the incidence of both primary and secondary endpoints and to identify the predictors for the composite primary end point, secondary end point, and each event including stroke/SE, major bleeding, new onset dementia, hospitalization for cardiac event and all‐cause mortality and to clarify the reality of AF management in Japan by using the data form the HAF‐NET Registry.

## 2 METHODS

### 2.1 Study cohort

The HAF‐NET Registry is multicenter, prospective, observational study of Japanese patients with AF. The patients were enrolled from April 2015 to August 2016. Inclusion criteria were those aged 20 or older in whom AF was diagnosed by a 12‐lead or Holter electrocardiogram. There were no exclusion criteria. A total of 22 institutions, all of which were located in Hyogo Prefecture, participated in this registry. They consisted of eight cardiovascular centers, two affiliated or community hospitals, and 12 primary care clinics. All patients were followed through a review of the inpatient and outpatient medical records, and additional information was obtained through contact with the patients, relatives, and/or referring physicians by mail or telephone. The data were checked by clinical research coordinators. The study protocol conformed to the ethical guidelines of the 1975 Declaration of Helsinki, CONSORT 2010 guidelines and was approved by the ethical committees of Kobe University (Committee of 2014.10.02., Approval No.1643). This study will be registered with the University Hospital Medical Information Network Clinical Trial Registry (UMIN‐CTR) (UMIN000036784), and the posted information will be updated as needed to reflect the protocol amendments and study progress.

### 2.2 Registration card and data collection

**Figure 1:**
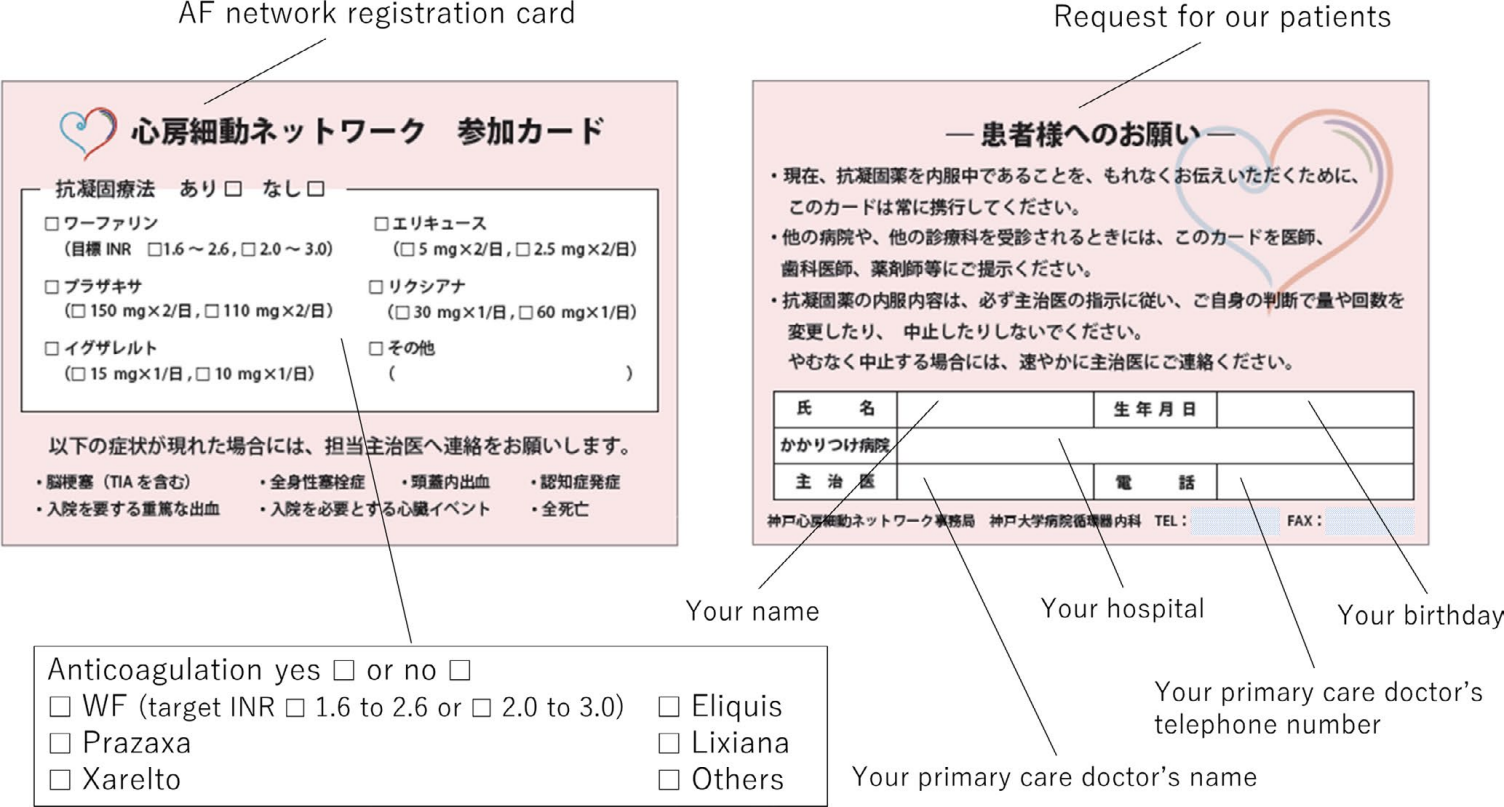
Registration card. The left panel shows the front side of the registration card where the actual anticoagulation therapy could be checked. The following text was described in the registration card: “Please inform your primary care doctor of the following events: Ischemic stroke, SE, Hemorrhagic stroke, new onset dementia, hospitalization for major bleeding, hospitalization for cardiac event, all‐cause mortality” was written by Japanese. The right panel shows the opposite side of the registration card where the patient name and birthday, primary care doctor's name and telephone number were written. The following text was described in the registration card: “Please always carry this card to inform your doctors of anticoagulation therapy. When you see a doctor, please show this card your doctors, dentists and pharmacists. According to the doctor's suggestion, please do not change the dosage of anticoagulants by self‐determination. Please inform your primary care doctor when the anticoagulation therapy reluctantly stopped.”

All patients had a certification of attendance, which contained information including the anticoagulation therapy regimen and contact information of the primary care doctor (Figure 1). Even though clinical adverse events occurred while being seen by secondary care doctors, they could inform the primary care doctor of the events by using this card. The primary care doctor was able to log into the website and register information about any adverse clinical events. The clinical patient data were registered on the online database system by the doctor in charge at each institution. The data were automatically checked for any missing or contradictory entries and values out of the normal range. Additional editing checks were performed by the clinical research coordinators at the general office of the registry.

The baseline clinical background data were as follows: patient clinical characteristics including the date of birth, age, gender, body weight, serum creatinine level, date when AF was diagnosed, history of treatment including CA, cardiac surgery, percutaneous coronary intervention, or coronary arterial bypass grafting, type of AF, comorbidities, and risk factors including heart failure, hypertension, diabetes mellitus, strokes/TIAs, vascular disease, valvular disease, ischemic heart disease, cardiomyopathy, dementia, whether patients smoked or consumed alcohol at the time of enrollment, a reduced left ventricular function (%FS < 25% or EF < 35%), current medications including anticoagulant drugs (DOACs or warfarin) and antiplatelet drugs, and subjective symptoms including palpitations, dyspnea and dizziness. Paroxysmal AF was defined as AF that terminated spontaneously within 7 days, while persistent AF was defined as AF that lasted for > 7 days but could be terminated with medication or electrical cardioversion. Long‐lasting persistent AF was defined as AF that lasted > 1 year.

The risk of a stroke was evaluated by the CHADS_2_ score and CHA_2_DS_2_‐VASc score.^13^ The risk of bleeding was evaluated by the HAS‐BLED score.^14^ A PT‐INR of 1.6‐2.6 was the optimal therapeutic range for patients aged 70 or older and a PT‐INR of 2.0‐3.0 was appropriate for patients aged 69 or younger.

### 2.3 Primary and secondary endpoints

The primary endpoints of this registry were symptomatic cerebral infarctions including TIAs, SEs, and fatal bleeding complications requiring hospitalization including an intracranial hemorrhage. A TIA was defined as a sudden onset of focal neurologic symptoms and/or a sign lasting less than 24 hours, brought on by a transient decrease in the blood flow, which rendered the brain ischemic in the area producing the symptom. Fatal bleeding complications were defined as a reduction in the hemoglobin level by ≥2 g/dL, a transfusion of ≥ 2 units of blood, or symptomatic bleeding in a critical area or organ, following the International Society on Thrombosis and Hemostasis definition. The secondary end points were a composite of new onset dementia, cardiac events requiring hospitalization, and all‐cause death. The diagnosis of dementia was based on the Mini‐Mental State Examination and/or Hasegawa dementia rating scale.

### 2.4 Statistical analysis

Continuous data were presented as the mean ± SD for normally distributed variables. Medians and quartiles were given for nonnormally distributed variables. If these data followed a normal distribution, they were tested with an unpaired t‐test or Welch test. If not, they were tested with a Mann‐Whitney test. Categorical variables were analyzed with the Fisher's exact test. Cox proportional hazards regression models were used to estimate the hazard ratios and 95% confidence intervals for each event. Previously reported variables including age, gender, BW, AF type, AFCA history, valvular disease, ischemic heart disease, cardiomyopathy, EF less than 35%, heart failure, hypertension, age > 75 years, diabetes mellitus, stroke/TIA, vascular disease, antiplatelet drug, DOAC use, and HAS‐BLED were also selected as cofounders. The multivariable Cox proportional hazards regression model included variables with a *P* < .05 using an unadjusted Cox proportional hazard regression analysis. To compare the clinical events between the warfarin and DOAC users, 667 age, BW, and CHADS_2_ score‐matched DOAC and warfarin users were tested. The cumulative incidence of a stroke or SE was determined by the Kaplan‐Meier method. The survival analysis between warfarin and DOACs was performed using a log‐rank test. A value of *P* < .05 was considered statistically significant. All statistical analyses were performed using SPSS, Release 25 software (SPSS).

## 3 RESULTS

A total of 2113 patients were enrolled from 38 institutions in Hyogo prefecture between April 2015 and August 2016. Of those, 1343 (64%) were enrolled from cardiovascular centers, 66 (3%) from affiliated or community hospitals, and 704 (33%) from private clinics. Two thousand and seventy (98%) of the 2113 patients were followed for 1 year after the enrollment and the mean follow‐up period was 355 ± 43 days.

### 3.1 Baseline characteristics of the registered patients

**Figure 2:**
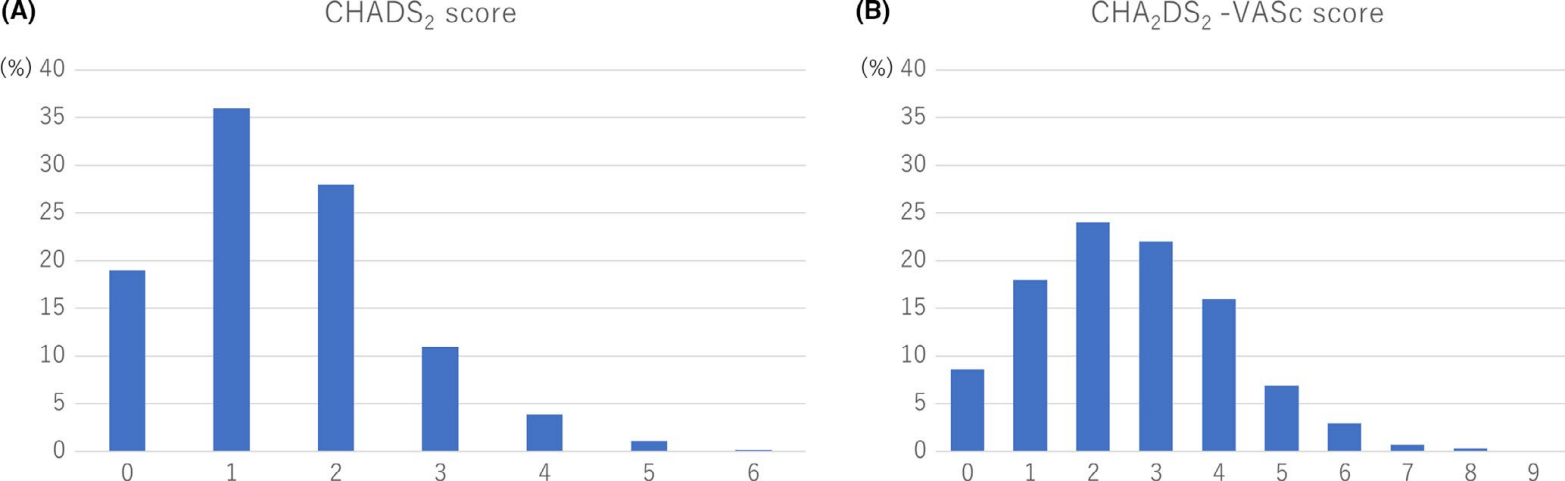
The distribution of the CHADS_2_ and CHA_2_DS_2_‐VASc scores. Panel A shows the CAHDS_2_ score distribution and panel B the CHA_2_DS_2_‐VASc score distribution

The baseline characteristics of the registered patients are summarized in Table 1. Almost 70% of the patients were male. The mean age was 70.1 years and 36% of them were aged 75 or over. Half of the patients had paroxysmal AF. Almost half of the patients were symptomatic and the most common subjective symptom was palpitations. The mean CHADS_2_ and CHA_2_DS_2_‐VASc scores were 1.5 ± 1.1 and 2.6 ± 1.6, respectively. Figure 2 shows the patients distribution according to the CHADS_2_ score and CHA_2_DS_2_‐VASc score. A CHADS_2_ score = 1 and CHA_2_DS_2_‐VASc score = 3 were the most common subpopulations. Table 2 shows the comorbidities of the patients. Hypertension was by far the most prevalent underlying disease, and 21.3% of the patients suffered from heart failure, of which 5.2% had an ejection fraction of <35%. Ischemic heart disease and valvular disease were present in 7.2% and 13.1%, respectively. Of those, mitral regurgitation was remarkably frequent. Of note, dementia was present in 2.7% of the patients. Almost 30% of the patients had a history of CA.

**Table 1:** Baseline characteristics of the patients

	Overall (n = 2113)	Warfarin (n = 725)	DOAC (n = 1118)	*P* value
Male (%)	1459 (69%)	493 (68%)	778 (70%)	.471
Weight (kg)	63.9 ± 12.7	62.5 ± 12.6	65.0 ± 12.7	<.001
Age (years old)	70.1 ± 10.8	73.1 ± 9.9	68.9 ± 10.5	<.001
20‐29	8 (0.4%)	0 (0%)	5 (0.4%)	
30‐39	17 (0.8%)	2 (0.3%)	9 (0.8%)	
40‐49	72 (3.4%)	14 (2%)	39 (3%)	
50‐59	225 (11%)	52 (7%)	129 (12%)	
60‐69	616 (29%)	165 (23%)	364 (33%)	
70‐79	782 (37%)	289 (40%)	419 (37%)	
80‐89	366 (17%)	191 (26%)	143 (13%)	
90‐99	27 (1.3%)	12 (2%)	10 (1%)	
≥75	761 (36%)	266 (37%)	379 (34%)	<.001
Range	20‐95	37‐94	20‐94	
Serum creatinine (mg/dL)	0.97 ± 0.65	1.07 ± 0.75	0.88 ± 0.36	<.001
Type of AF				<.001
Paroxysmal AF	1,066 (50%)	273 (38%)	595 (53%)	
Persistent AF	546 (26%)	180 (25%)	319 (29%)	
Long lasting persistent AF	501 (24%)	272 (37%)	204 (18%)	
Symptomatic	1,136 (54%)	351 (48%)	624 (56%)	.002
Palpitation	916 (43%)	255 (35%)	522 (47%)	<.001
Shortness of breath	341 (16%)	163 (22%)	160 (14%)	<.001
General fatigue	102 (5%)	50 (7%)	47 (4%)	.014
Dizziness	40 (2%)	14 (2%)	18 (2%)	.590

Note

Values are presented as the mean ± SD or n (%).

Abbreviations: AF, atrial fibrillation; DOAC, direct oral anticoagulant.

**Table 2:** Comorbidities

	Overall (n = 2113)	Warfarin (n = 725)	DOAC (n = 1118)	*P* value
Stroke/TIA	196 (9.3%)	82 (11%)	106 (10%)	.208
Heart failure	463 (22%)	244 (34%)	193 (17%)	<.001
EF < 35%	110 (5.2%)	73 (10%)	34 (3%)	<.001
Valvular disease	284 (13%)	180 (25%)	86 (8%)	<.001
Aortic regurgitation	77 (3.6%)	44 (6%)	25 (2%)	<.001
Mitral regurgitation	173 (8.2%)	106 (15%)	57 (5%)	<.001
Tricuspid regurgitation	63 (3.0%)	47 (6%)	12 (1%)	<.001
Aortic stenosis	40 (1.9%)	27 (4%)	7 (0.6%)	<.001
Mitral stenosis	28 (1.3%)	26 (4%)	2 (0.2%)	<.001
Ischemic heart disease	153 (7.2%)	75 (10%)	66 (6%)	.001
Cardiomyopathy	178 (8.4%)	84 (12%)	83 (7%)	.003
Vascular disease	130 (6.2%)	53 (7%)	66 (6%)	.245
Diabetes mellitus	328 (16%)	133 (18%)	163 (15%)	.032
Hypertension	1,208 (57%)	423 (58%)	663 (59%)	.698
Renal disease	242 (11%)	137 (19%)	77 (7%)	<.001
Hemodialysis	11 (0.5%)	4 (0.6%)	0 (0%)	.024
Liver disease	31 (1.5%)	6 (0.8%)	22 (2%)	.053
Dementia	56 (2.7%)	28 (4%)	22 (2%)	.009
History of open‐heart surgery	211 (10%)	133 (18%)	64 (6%)	<.001
History of CABG	35 (1.7%)	23 (3%)	9 (0.8%)	<.001
History of PCI	116 (5.5%)	55 (8%)	54 (5%)	.015
History of CA for AF	614 (29%)	163 (22%)	277 (25%)	.264
Pacemaker implantation	114 (5.4%)	66 (0.8%)	40 (4%)	<.001
CHADS_2_ score	1.5 ± 1.1	1.8 ± 1.2	1.4 ± 1.1	<.001
CHA_2_DS_2_‐VASc score	2.6 ± 1.6	3.0 ± 1.6	2.5 ± 1.5	<.001
HAS‐BLED score	1.3 ± 1.0	1.6 ± 1.0	1.2 ± 0.9	<.001

Note

Values are presented as the n (%). Vascular disease indicates patients with a prior myocardial infarction, peripheral arterial disease, or aortic plaque. Renal disease indicates the patients with hemodialysis, transplantations, or abnormalities of the creatinine level of >2.26 mg/dL or >200 μmol/L. Liver disease indicates patients with cirrhosis or a bilirubin level > 2×normal with an AST/ALT/AP >3 × normal.

Abbreviations: AF, atrial fibrillation; CABG, coronary arterial bypass grafting; EF, ejection fraction; TIA, transient ischemic attack; PCI, percutaneous coronary intervention.

Compared to the patients without a CA history, those with a history of CA had a younger age, mainly paroxysmal AF, fewer underlying diseases, lower CHADS_2_ and CHA_2_DS_2_‐VASc scores, and lower HAS‐BLED score (age: 65.7 ± 10.3 vs. 71.8 ± 10.6 years old, *P* < .001; heart failure: 15.3% vs. 24.6%, *P* < .001; CHADS_2_ score: 1.23 ± 1.12 vs. 1.59 ± 1.11, *P* < .001; CHA_2_DS_2_‐VASc score: 2.23 ± 1.68 vs. 2.73 ± 1.50, *P* < .001; HAS‐BLED score: 1.08 ± 0.97 vs. 1.40 ± 0.97, *P* < .001), while a stroke/TIA history was more frequently observed in those with an AFCA history (11.4% vs. 8.4%, *P* = .007). Of note, fewer anticoagulant drugs or antiplatelet drugs were used in the patients with an AFCA history (anticoagulant drug use: 71.5% vs. 93.6%, *P* < .001; antiplatelet drug use: 5.9% vs. 10.6%, *P* = .001).

### 3.2 Medications in HAF‐NET patients

**Figure 3:**
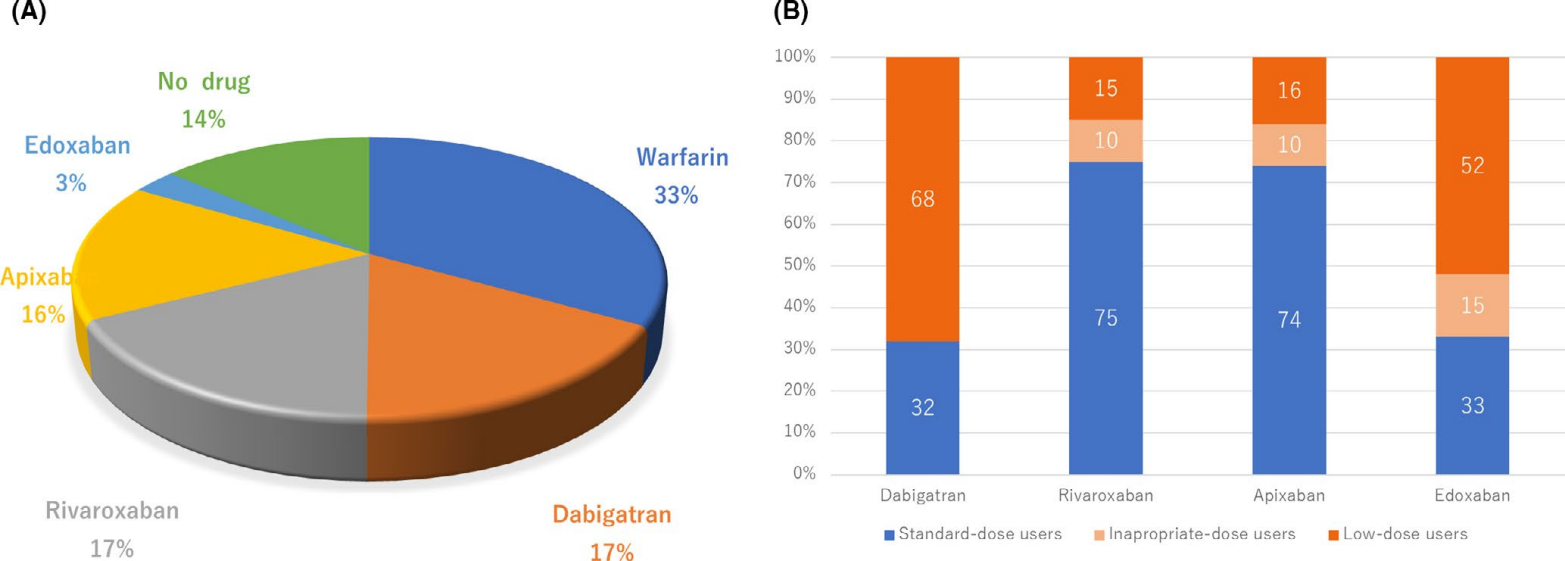
The proportion of anticoagulants and dosing. Panel A shows the proportion of anticoagulants and panel B the proportion of standard or low dosing of each DOAC. The numbers indicated the percentages

Figure 3A showed the medications at enrollment. Anticoagulant drugs were used in 86% of the patients. DOACs were prescribed in almost half of the patients. Edoxaban was prescribed in a small number of the patients, because the enrollment of this study ended several months after edoxaban was released. Antiplatelet drugs were used for 9.2% of the patients. Figure 3B shows the distribution of each DOAC dose. Low‐dose users were common in both the dabigatran and edoxaban users, but not in the rivaroxaban and apixaban users. Of note, inadequate dose user was extremely low in DOAC group (10% in Rivaroxaban users, 10% in Apixaban users and 15 % in Edoxaban users).

Compared to the DOACs, warfarin was prescribed in elderly patients with a lower BW and higher creatinine level (age: 73.1 ± 9.9 vs. 68.9 ± 10.5 years old, *P* < .001; BW: 62.5 ± 12.6 vs. 65.0 ± 12.7, *P* < .001; serum creatinine level: 1.07 ± 0.75 vs. 0.88 ± 0.36, *P* < .001). The AF types including paroxysmal, persistent, and long‐lasting AF were equally distributed in the warfarin users, while paroxysmal AF was dominantly found in the DOAC users. The symptoms were less in the warfarin users (Table 1). Heart failure, an EF of <35%, valvular disease, ischemic heart disease, cardiomyopathy, diabetes mellitus, renal disease, hemodialysis, dementia, a history of open‐heart surgery, a CABG, and PCI were frequently observed in the warfarin users, which resulted in a higher CHADS_2_. CHA_2_DS_2_‐VASc and HAS‐BLED score as compared to the DOACs users (CHADS_2_ score: 1.8 ± 1.2 vs. 1.4 ± 1.1, *P* < .001; CHA_2_DS_2_‐VASc score: 3.0 ± 1.6 vs. 2.5 ± 1.5, *P* < .001, HAS‐BLED score: 1.6 ± 1.0 vs. 1.2 ± 0.9, *P* < .001; Table 2).

No anticoagulant therapy was performed in 270 (13%) patients who were significantly younger and their CHADS_2_ and CHA_2_DS_2_‐VASc scores were significantly lower (age: 66.2 ± 12.6 vs. 70.6 ± 10.4 years old, *P* < .001; CHADS_2_ score: 1.0 ± 0.9 vs. 1.6 ± 1.1, *P* < .001; CHA_2_DS_2_‐VASc score: 1.9 ± 1.5 vs. 2.7 ± 1.6, *P* < .001). Of note, a CA history was more frequently observed in patients without anticoagulant therapy than in those who did not undergo CA (174 [64%] of 270 vs. 440 [24%] of 1843 patients, *P* < .001).

### 3.3 The incidence of primary and secondary endpoints and the clinical predictors

A composite of the primary and secondary endpoints was found in 30 (1.4%) and 99 (4.4%) patients, respectively. Of those, ischemic strokes/SEs and major bleeding occurred in 13 (0.6%) and 17 (0.8%) patients, respectively. New onset dementia, hospitalization for cardiac events, and all‐cause death occurred in eight (0.4%), 60 (2.8%), and 29 (1.4%) patients, respectively. According to a multivariable Cox proportional hazard regression analysis, the most predictive model of a stroke/SE consisted of the AF type (persistent) and that of major bleeding consisted of the BW as well as the HAS‐BLED score (AF type: HR: 9.54, 95% CI: 1.23‐73.97, *P* = .03; BW: HR: 0.94, 95% CI: 0.90‐0.99, *P* = .03; HAS‐BLED score: HR: 1.82, 95% CI: 1.12‐2.96, *P* = .02; Table 3). The most predictive model of new onset dementia consisted of the age and diabetes mellitus (age: HR: 1.12, 95% CI: 1.01‐1.24, *P* = .03; diabetes mellitus: HR: 3.51, 95% CI: 1.17‐10.54, *P* = .03), that of hospitalization for cardiac events consisted of AFCA history as well as serum creatinine, heart failure (AFCA history: hazard ratio, 0.44; 95%CI, 0.20‐0.99; *P* = .04; serum creatinine: HR: 1.25, 95% CI: 1.02‐1.53, *P* = .03; heart failure: HR: 2.12, 95% CI: 1.12‐4.00, *P* = .02), and that of all‐cause mortality consisted of the age and EF less than 35% (age: HR: 1.21, 95% CI: 1.12‐1.32, *P* < .01; EF less than 35%: HR: 5.71, 95% CI: 1.68‐19.42, *P* < .01; Table 4).

**Table 3:** Cox regression analysis of the primary endpoint and clinical characteristics

	Stroke or SE		Major bleeding		Composite primary endpoint	
	Adjusted HR	*P* value	Adjusted HR	*P* value	Adjusted HR	*P* value
Age (years old)	1.07 (1.00‐1.14)	.06			1.01 (0.94‐1.10)	.72
BW (kg)			0.94 (0.90‐0.99)	.03	0.97 (0.93‐1.00)	.08
AF type (persistent)	9.54 (1.23‐73.97)	.03			1.89 (0.75‐4.74)	.18
AF ablation history					0.24 (0.05‐1.09)	.06
Valvular disease			1.65 (0.54‐4.99)	.38	2.06 (0.86‐4.95)	.11
Ischemic heart disease			3.23 (0.81‐12.94)	.10	2.46 (0.77‐7.90)	.13
Cardiomyopathy					1.25 (0.35‐4.52)	.73
EF < 35%			1.79 (0.50‐6.50)	.37	2.03 (0.58‐7.15)	.27
Heart failure			2.76 (0.81‐9.41)	2.76	1.78 (0.70‐4.40)	.23
Age > 75 years					0.71 (0.21‐2.41)	.59
Diabetes mellitus	3.51 (1.17‐10.54)	.03			3.05 (1.32‐7.03)	<.01
Stroke/TIA					2.26 (0.72‐7.10)	.16
Vascular disease	3.41 (0.92‐12.65)	.07			1.29 (0.35‐4.74)	.70
Antiplatelet drugs			0.88 (0.21‐3.63)	.85	1.01 (0.30‐3.43)	.99
HAS‐BLED score			1.82 (1.12‐2.96)	.02		

Abbreviations: AF, atrial fibrillation; BW, body weight; EF, ejection fraction; SE, systemic embolism; TIA, transient ischemic attack.

**Table 4:** Cox regression analysis of the secondary endpoint and clinical characteristics

	New onset dementia		Hospitalization for cardiac event		All‐cause mortality		Composite secondary endpoint	
	Adjusted HR	*P* value	Adjusted HR	*P* value	Adjusted HR	*P* value	Adjusted HR	*P* value
Age (years old)	1.12 (1.01‐1.24)	.03	1.70 (0.94‐3.12)	.08	1.21 (1.12‐1.32)	<.01	1.04 (1.00‐1.08)	.06
BW (kg)					0.99 (0.95‐1.03)	.59	1.00 (0.98‐1.02)	.65
AF type (persistent)					0.59 (0.27‐1.33)	.21	1.11 (0.69‐1.80)	.66
Serum creatinine			1.25 (1.02‐1.53)	.03			1.20 (1.00‐1.43)	.05
AF ablation history			0.44 (0.20‐0.99)	.04	0.25 (0.03‐1.94)	.18	0.50 (0.25‐0.99)	.04
Valvular disease	1.70 (0.35‐8.30)	.52	2.27 (1.22‐4.22)	<.01	1.68 (0.70‐4.03)	.24	1.68 (1.01‐2.78)	.05
Ischemic heart disease			1.73 (0.71‐4.22)	.23	0.82 (0.22‐3.10)	.77	1.23 (0.57‐2.66)	.59
Cardiomyopathy					1.49 (0.14‐15.48)	.74		
EF < 35%			2.04 (0.27‐15.14)	.90	5.71 (1.68‐19.42)	<.01	2.65 (0.63‐11.20)	.18
Heart failure	4.92 (0.86‐28.19)	.07	2.12 (1.12‐4.00)	.02	2.37 (0.94‐5.96)	.07	2.24 (1.35‐3.73)	<.01
Age > 75 years					0.51 (0.12‐2.21)	.37	1.15 (0.58‐2.29)	.70
Diabetes mellitus	3.51 (1.17‐10.54)	.03			1.34 (0.48‐3.71)	.57	0.88 (0.48‐1.62)	.70
Stroke/TIA					1.73 (0.55‐5.43)	.34		
Vascular disease	3.41 (0.92‐12.65)	.07	1.98 (0.79‐4.97)	.14	3.18 (0.92‐10.94)	.07	2.31(1.13‐4.75)	.02
Antiplatelet drugs			1.44 (0.56‐3.67)	.45	1.22 (0.38‐3.95)	.74	1.22 (0.59‐2.50)	.59

Abbreviations: AF; atrial fibrillation; BW; body weight; EF; ejection fraction; SE; systemic embolism; TIA; transient ischemic attack.

Focusing on the patients without anticoagulation therapy, a composite primary and secondary endpoints was found in two (0.7%) and 11 (4.1%) of 270 patients, respectively. Of those, ischemic strokes/SEs and major bleeding occurred in one (0.4%) and one (0.4%) patients, respectively. New onset of dementia, hospitalization for cardiac event and all‐cause death occurred in one (0.4%), six (2.2%), and five (1.9%) patients, respectively. Notably, 174 (64%) of 270 patients without anticoagulation therapy had CA history. Although hospitalization for cardiac event was found in four (2%) of 174 patients, no other events including stroke/SE, major bleeding, new onset of dementia and all‐cause death were found in the 174 patients with CA. Furthermore, 614 (29%) of 2113 patients had CA history regardless of anticoagulation therapy, primary and secondary endpoints were found in 4(0.7%) and 18(3%), respectively. Of those, stroke/SE, major bleeding, new onset of dementia, hospitalization for cardiac event, and all‐cause death were found in one (0.2%), three (0.5%), two (0.3%), 15 (2%) and one (0.2%) patients, respectively.

**Figure 4:**
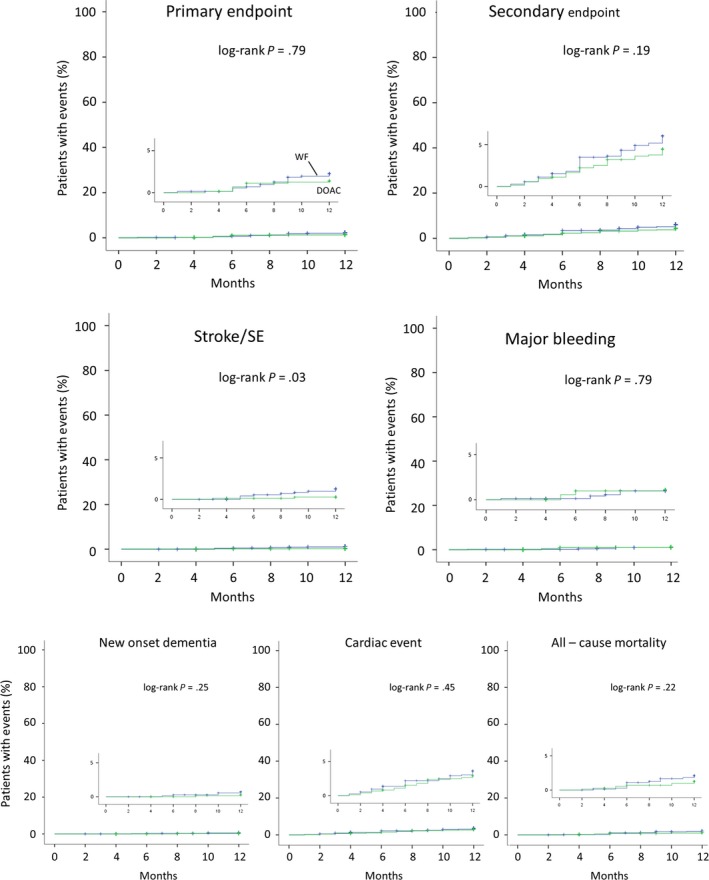
Kaplan‐Meier curve for the primary and secondary endpoints, ischemic strokes/SEs, major bleeding, new onset dementia, cardiac events, and all‐cause mortality between the warfarin and DOAC users. The clinical events were assessed among 667 age, BW, CHADS_2_ score, and CHA_2_DS_2_‐VASc score‐matched DOAC and warfarin users

To assess the clinical impact of the DOACs, 667 age, BW, CHADS_2_ score‐matched DOAC and warfarin users were compared (DOAC vs. warfarin; age: 72.0 ± 9.3 vs. 72.2 ± 9.7, *P* = .67; BW: 62.2 ± 11.5 vs. 62.9 ± 12.7, *P* = .30; CHADS_2_ score: 1.7 ± 1.1 vs. 1.7 ± 1.1, *P* = .96). A composite of the primary and secondary endpoints was found in nine (1.3%) and 24 (3.6%) DOAC users, and in 15 (2.2%) and 36 (5.4%) of the 667 warfarin users, respectively. Of those, the incidence of the ischemic strokes/SEs was significant less in DOAC users (1 [0.1%] of 667 vs. 8 [1.2%] of 667, log‐rank *P* = 0.02; Figure 4). The incidence of the other endpoints including major bleeding, new onset dementia, hospitalization for cardiac events, and all‐cause death did not differ between the DOAC and warfarin users (major bleeding: 8 [1.2%] vs. 7 [1.0%] of 667 patients, *P* = .79; new onset of dementia: 1 [0.1%] vs. 3 [0.4%] of 667 patients, *P* = .32; hospitalization for cardiac event: 16 [2.4%] vs. 23 [3.4%] of 667 patients, *P* = .26; all‐cause death: 7 [1.0%] vs. 12 [1.8%] of 667 patients, *P* = .25).

## 4 DISCUSSION

### 4.1 Main findings of the study

The data from the HAF‐NET registry demonstrated a higher DOAC use and AFCA history as compared to the previous studies, which resulted in excellent outcomes after 1 year of follow‐up among Japanese patients with AF in the DOAC and AF ablation era. Persistent AF and a lower BW were strongly associated with stokes/SEs and major bleeding, respectively. AFCA history as well as age and heart failure were associated with the composite secondary endpoints including new onset dementia, hospitalization for cardiac events, and all‐cause mortality.

### 4.2 Patient characteristics

One‐third (800 patients) of the patients in this registry were from Chuo‐Ku, which is located in the southern region of Kobe city. The population of Chuo‐Ku is approximately 135 000 people. Based on the epidemiological prevalence of AF in the Japanese population of 0.6%, the number of AF patients in Chuo‐Ku was estimated to be approximately 810. As the number in our registry was almost equal to the estimated AF patients in Chuo‐Ku, the AF patents in this registry were assumed to fully reflect a typical ward in Kobe city.

In Japan, real‐world data of the anticoagulation therapy in patients with AF have been published from two major AF registry such as FUSHIMI and SAKURA registry.^15^, ^16^ The enrollment of the FUSHIMI and SAKURA registries was performed from 2011 to 2014 and from 2013 to 2015, respectively. As compared to the patient characteristics of the patients enrolled by the FUSHIMI and SAKURA registries, younger patients with lower CHADS_2_ scores were enrolled in our registry. The mean age of the HAF‐NET, SAKURA, and FUSHIMI registries was 70, 72, and 74 years old, respectively. The mean BW was 64, 64, and 58 kg and persistent AF 49%, 63% and 54% in the enrolled patients, respectively. The prevalence of a stroke/TIA history was 9%, 11%, and 19 %, respectively. The mean CHADS_2_ scores were 1.5, 1.8, and 2.1, respectively.

The FUSHIMI registry recruited patients mainly from private clinics, while the HAF‐NET and SAKURA registries recruited those from cardiovascular centers, affiliated hospitals, community hospitals, and private clinics. Sixty‐four and 48 private clinics participated in the FUSHIMI and SAKURA registries, respectively, while there were only 15 in the HAF‐NET registry. The proportion of patients from private clinics completely differed. This might be the reason why the patient characteristics differed among the three registries.

### 4.3 Medications in HAF‐NET patients

Warfarin was prescribed in only around 30% of the patients in the HAF‐NET registry. As compared to the FUSHIMI and SAKURA registries, the proportion of warfarin users was significantly smaller in the HAF‐NET registry (32% vs. 54% vs. 48% in the HAF‐NET, SAKURA, and FUSHIMI registries). However, the proportion of DOAC users was significantly greater (53% vs. 46% vs. 2% in the HAF‐NET, SAKURA, and FUSHIMI registries). Several studies from the J‐RHYTHM and SHINKEN databases reported the increase in DOAC users, from 6.1% in 2012 to 20.4 % in 2014, and from none in 2007‐2009 to 25.5% in 2010‐2012.^17^, ^18^ The HAF‐NET registry demonstrated the actual anticoagulation therapy use in the DOAC era. The prevalence of a low‐dose usage of DOACs was significantly less in rivaroxaban/apixaban users than in dabigatran/edoxaban users. Of importance, the SAKURA registry identified inappropriately low dosing in 19.7 to 27.6% of the DOAC users. The proportion of an adjusted low dosing was estimated to be almost 20% in the rivaroxaban or apixaban users and almost 50% in the dabigatran or edoxaban users. Furthermore, postmarketing studies for each DOAC also estimated that the proportion of an adjusted low dosing was almost 30% in the rivaroxaban or apixaban users and almost 60% in the dabigatran or edoxaban users.^19^, ^20^, ^21^, ^22^ The proportion of this estimated adequately low‐dosing usage in the SAKURA registry was similar to our results. This indicated that the inadequate low dosing in the HAF‐NET registry was extremely less than that in the previous AF registries. Almost 8 years have passed since dabigatran was released as the first DOAC in 2011. Over the past decade, we have experienced the importance of adequate dosing of DOACs, which has increased major bleeding as well as strokes or TIAs. Therefore, adequate dosing might be challenged in the recent real world of AF anticoagulation therapy in Japan. Actually, our data clearly supported this challenge and demonstrated excellent outcomes.

### 4.4 Primary and secondary endpoints and clinical predictors

The two major postmarket surveillance (PMS) studies (J‐Dabigatran surveillance, XAPASS) showed the incidence rates of major bleeding and thromboembolic events, suggesting that dabigatran and rivaroxaban were safe and effective in the Japanese clinical practice. The J‐Dabigatran surveillance demonstrated that major bleeding, strokes/SEs, and all‐cause death occurred in 1.1%, 1.2%, and 1.3%/year, respectively.^19^ The XAPASS study demonstrated that major bleeding, strokes/SEs, and all‐cause death occurred in 1.5%, 1.1%, and 2.1%/year, respectively.^23^ While the HAF‐NET registry demonstrated that major bleeding, strokes/SEs, and all‐cause death occurred in 0.6%, 0.8%, and 1.4%/year, which was likely better than that in the two major PMS studies. Although 13% of the patients without anticoagulation therapy and 30% of warfarin users were enrolled in the HAF‐NET registry, the CHADS_2_ score was lower and a CA history was found in almost 30% of the patients who mainly had a stroke history or no anticoagulation therapy. This might have reduced the AF burden, which resulted in an excellent outcome. Iguchi et al. demonstrated that patients with preexisting heart failure had higher unadjusted rates of strokes/SEs (HR, 1.40; 95% CI, 1.05‐1.85; *P* = .02 by a log‐rank test) as well as higher incidences of all‐cause death and a composite of all‐cause death or strokes/SEs.^24^ Although AFCA history could not be associated with strokes/SEs in the HAF‐NET registry, we could clearly demonstrate that the AFCA history could be associated with the lower cardiovascular hospitalization, which indicated that the possibility improving the higher strokes/SEs as well as higher incidences of all‐cause death and a composite of all‐cause death or strokes/SEs. Recently, Packer et al reported that the composite endpoints of mortality and cardiovascular hospitalization exhibited a significant 17% relative lower event rate for the CA group than medical therapy group. To improve the mortality as well as quality of life, CA might be aggressively considered especially in patients with heart failure or a stroke history.^12^

### 4.5 Catheter ablation and anticoagulation therapy

Recently, several studies have reported the impact of CA on the mortality and cardiovascular hospitalization in patients with AF. Especially in patients with heart failure, the impact has been greater. The CATSLE‐AF study clearly demonstrated that CA was associated with a significantly lower rate of the composite end point of death from any cause or hospitalization for worsening heart failure as compared to medical therapy. Although no statistical significance could be found, cerebrovascular accidents were dramatically reduced by CA as compared to medical therapy.^11^ Furthermore, the impact of CA has been greater in patients with an age of <65 years old, heart failure of <NYHA functional class II, and EF of ≧ 25%. The CABANA study also reported that the impact of CA was greater in patients with an age of <65 years old.^12^ Those two RCTs indicated the importance of early intervention to maintain sinus rhythm by CA in patients with AF. Patients with a CA history in the HAF‐NET registry had relatively low CHADS_2_ and CHA_2_DS_2_‐VASc scores and the incidence of clinical events was extremely low. This indicated that CA could be considered as an early intervention for AF management in the real world. Based on those clinical results, the Japanese guidelines have been updated and the latest one (2018 JCS/JHRS Guideline on Non‐Pharmacotherapy of Cardiac Arrhythmias) was published in May 2019. In the latest guidelines, CA was recommended as a class IIa indication for drug refractory, recurrent paroxysmal and persistent AF regardless of heart failure. This will facilitate the CA in patients with heart failure, which might strengthen the impact of CA on AF management in the next decade.

### 4.6 Impact of DOACs on preventing clinical events

Previous RCTs have revealed better clinical outcomes especially for fatal bleeding under DOAC therapy than under warfarin therapy. However, fewer Japanese patients could be enrolled in those RCTs.^5^, ^6^, ^7^, ^8^ The SAKURA registry showed no significant differences in the rates of strokes or SEs, major bleeding, and all‐cause mortality for DOAC vs. warfarin users. Under propensity score matching, the incidence of strokes or SEs and all‐cause death remained equivalent, but the incidence of major bleeding was significantly lower among DOAC than warfarin users.^25^ In the HAF‐NET registry, the incidence of strokes or SEs was significantly lesser in the DOAC users, but not that for major bleeding. This discrepancy might be caused by the frequency of the CA history. Progression of AF was reported to be associated with an increased risk of clinical adverse events during the arrhythmia progression period from paroxysmal to persistent AF among Japanese patients with AF. The risk of adverse events was also transiently elevated during the progression period from paroxysmal to persistent AF and declined to a level equivalent to persistent AF after the progression.^26^ A CA history was found in almost 10% and 25% in the SAKURA an HAF‐NET registries, respectively. As compared to medical therapy, CA could strongly reduce the AF burden and no progression toward persistent AF was observed during a median follow‐up of 6 years especially in patients with paroxysmal AF.^27^ In such patients without AF recurrence after a successful CA, DOACs might be continued without a dose reduction, while the PT‐INR level might be controlled at a lower level to avoid the fatal bleeding. This suggested the importance of CA and DOACs for preventing strokes or SEs and the awareness of an adequate DOAC lower dosing after a successful CA.

### 4.7 Dementia and AF

A meta‐analysis reported that AF was independently associated with an increased risk of all forms of dementia.^1^ The incidence of dementia in the patients without AF was almost 3.0% during a follow‐up period of over 5 years. After a dementia diagnosis, the presence of AF was associated with a marked increased risk of mortality.^2^ Recently, individuals with AF have been reported to have an almost threefold increased risk of dementia during a 12 year follow‐up (HR 2.8; 95% CI 1.3‐5.7; *P* = .004). The population attributable risk for dementia resulting from AF was 13%. They concluded that patients with AF should be screened for cognitive symptoms.^28^ In the HAF‐NET registry, dementia diagnosed before enrollment was found in 56 (2.7%) of the patients and the incidence of new onset dementia was 8 (0.4%) patients. This annulus incidence of dementia was similar to that in patients without AF. This might be the impact from anticoagulation therapy with DOACs and a strong rhythm control therapy with CA. We hope that this impact would continue during the follow‐up of over 3 years because the average time to the development of dementia has been reported to be almost 3 years.

### 4.8 Study limitations

This study had several limitations. First, this study was designed as a prospective observational study, therefore, only associations were shown, not causality. The possibility of unmeasured or residual confounding factors was not ruled out. Second, anticoagulant therapy was assessed at the time of the enrollment, but the changes in the medical therapy could not be assessed. Third, to assess the impact of the DOAC therapy, age, BW, and CHADS_2_ score‐matched DOAC, and warfarin users were compared because of the small number in each medical therapy group. Fourth, this study involved AF patients recruited from a small region of Japan, and therefore, the results might not be generalizable to the overall population.

## 5 CONCLUSION

The HAF‐NET registry was characterized as (a) having a high incidence of DOAC prescriptions and a CA history and (b) including relatively younger patients with lower CHADS_2_ scores. In the DOAC and CA era, the incidence of ischemic strokes/SEs, major bleeding and hospitalization for the cardiac events could be strongly reduced in patients with AF. However, some unsolved issues of AF management still remain especially in elderly patients with persistent AF and a low BW.

## CONFLICT OF INTERESTS

The Section of Arrhythmia is supported by an endowment from Medtronic JAPAN and Abbott JAPAN. The authors have reported that they have no relationship relevant to the contents of this paper to disclose.
