# Supplementary material for: One‐year clinical outcomes of anticoagulation therapy among Japanese patients with atrial fibrillation: The Hyogo AF Network (HAF‐NET) Registry
Source: J Arrhythm. 2019 Aug 16;35(5):697–708. doi: 10.1002/joa3.12226 (PMC6786980; doi:10.1002/joa3.12226)
Supplement: Supplementary file 1 [file JOA3-35-697-s001.docx]

**Appendix**

The key personnel and institutions participating in the registry are as follows:

Chief investigator: Yoshida A (Kita-harima Medical Center)

Vice-chief investigator: Fukuzawa K (Section of Arrhythmia, Division of Cardiovascular Medicine, Department of Internal Medicine, Kobe University Hospital)

Steering Committee: Yoshida A (Kita-harima Medical Center), Takeuchi M (Kobe Medical Association), Fukuzawa K (Section of Arrhythmia, Division of Cardiovascular Medicine, Department of Internal Medicine, Kobe University Hospital)

Statistical Analysis: Kiuchi K (Section of Arrhythmia, Division of Cardiovascular Medicine, Department of Internal Medicine, Kobe University Hospital)

Participating institutions: Section of Arrhythmia, Division of Cardiovascular Medicine, Department of Internal Medicine, Kobe University Hospital (Hyogo K, Kiuchi K, Fukuzawa K, Takami M, Yamashita S, Matsumoto A, Ichibori H, Konishi H, Imada H, Kurose J, Nagamatsu Y, Suehiro H, Akita T, Takemoto M, Nakamura T, Sakai J); Department of Internal Medicine, Kobe University Hospital (Shinke T, Otake H, Hirata K); Kita-harima Medical Center (Yoshida A, Awano K, Ohashi Y, Yamada S, Nakagawa M, Yamawaki K, Tagashira T, Hiraishi M, Nakabayashi A, Ishii T, Hamaguchi H, Oda T, Takada M, Nagata K, Takami K, Tsuda S); Takeuchi Clinic (Takeuchi M); Himeji Cardiovascular Center (Shimane A); Kakogawa Central City Hospital (Okajima K, Shimizu H, Onishi Y, Nakanishi T, Nakamura H, Kadotani M, Yasuda T, Miwa K, Kaetsu Y, Yatomi A, Matsuoka Y, Nakaoka H, Yamana S, Fujinami Y, Shimoura H, Shiraki R, Namura H); Aijinkai Healthcare Corporation Akashi (Sakamoto S); Kobe City Medical Center General Hospital (Kobori A, Furukawa Y, Sasaki Y); Kobe Century Memorial Hospital (Sano H, Suematsu M, Mataki H, Mizutani K, Masuda Y); Japanese Red Cross Kobe Hospital (Doi T); Ichikawa Internal Medicine Cardiology (Ichikawa Y); Odake Internal Medicine Cardiology (Odake M); Yano Internal medicine clinic (Yano T); Okukubo Clinic (Okukubo T); Kudo Internal medicine clinic (Kudo Y); Shima Internal medicine clinic (Shima T); Ooyama kinen Hospital (Sekiya J); Okada Clinic (Okada T); Tamada Internal medicine (Tamada K); Kawahara Internal Medicine (Kawahara Y); Tanaka Internal Medicine Cardiology (Tanaka C); Tabuchi Clinic (Tabuchi H)
